# Executive Functioning in Men with Schizophrenia and Substance Use Disorders. Influence of Lifetime Suicide Attempts

**DOI:** 10.1371/journal.pone.0169943

**Published:** 2017-01-18

**Authors:** Ana Adan, Maria del Mar Capella, Gemma Prat, Diego A. Forero, Silvia López-Vera, José Francisco Navarro

**Affiliations:** 1 Department of Clinical Psychology and Psychobiology, Faculty of Psychology, University of Barcelona, Barcelona, Spain; 2 Institute of Neurosciences, University of Barcelona, Barcelona, Spain; 3 Laboratory of NeuroPsychiatric Genetics, Biomedical Sciences Research Group, School of Medicine, Universidad Antonio Nariño. Bogotá, Colombia; 4 Department of Psychobiology, School of Psychology, University of Málaga, Málaga, Spain; University of Granada, SPAIN

## Abstract

**Background:**

Lifetime suicide attempts in patients with comorbidity between psychotic disorders and Substance Use Disorder (SUD), known as dual diagnosis, was associated with a worse clinical and cognitive state, poor prognosis and premature death. However, to date no previous study has examined the cognitive performance of these patients considering as independent the presence or absence of lifetime suicide attempts.

**Methods:**

We explore executive functioning differences between suicide attempters and non-attempters in dual schizophrenia (DS) patients and the possible related factors for both executive performance and current suicide risk. Fifty DS male patients in remission of SUD and clinically stables, 24 with and 26 without lifetime suicide attempts, were evaluated. We considered Z scores for all neuropsychological tests and a composite summary score for both premorbid IQ and executive functioning.

**Results:**

DS patients showed low performance in set-shifting, planning and problem solving tasks. Those with suicide attempts presented lower composite summary scores, together with worse problem solving skills and decision-making, compared with non-attempters. However, after controlling for alcohol dependence, only differences in decision-making remained. Executive functioning was related to the premorbid intelligence quotient, and several clinical variables (duration, severity, months of abstinence and relapses of SUD, global functioning and negative symptoms). A relationship between current suicide risk, and first-degree relatives with SUD, insight and positive symptoms was also found.

**Conclusions:**

Our results suggest that problem solving and, especially, decision-making tasks might be sensitive to cognitive impairment of DS patients related to presence of lifetime suicide attempts. The assessment of these executive functions and cognitive remediation therapy when necessary could be beneficial for the effectiveness of treatment in patients with DS. However, further research is needed to expand our findings and overcome some limitations of this study.

## Introduction

Substance Use Disorder (SUD) is becoming more prevalent over the years and it is highly common in Schizophrenia (SZ), a comorbidity known as Dual Schizophrenia (DS). This dual diagnosis has relevant clinical implications, given that substance use could exacerbate the SZ symptoms [1] as well as the cognitive impairment [2, 3], it also worsens the prognosis of the disorder [4, 5], and increases the risk of suicide [6]. Thus, it is necessary to study new ways to improve the health-related quality of life and reducing mortality of DS patients. Given that suicide is one of the main causes of premature death in SZ [7, 8], preventing suicide and diminishing its risk factors deserve great consideration as main targets of care, especially in the DS population.

Around 20–50% of SZ patients will attempt suicide in their lifetime [9, 10]. The suicide risk is greater in first or acute psychotic episodes, during the first six months after hospitalization [11, 12] and in SUD comorbid conditions [6, 8, 11, 13–16]. Alcohol is one of the most studied substances since it has been shown to have a significant impact on suicide [17–19] and cognition [20, 21]. Furthermore, DS patients, compared to SUD patients, are less motivated to change their consumption pattern, are more difficult to involve in treatment, exhibit slower progress, tend to give up long-term programs [22] and remain at risk of relapse even years after a period of full remission [23].

In addition to the SUD, other risk factors for suicide described in SZ patients are: male gender, single marital status, living alone and social isolation, family or personal history of lifetime suicide attempts, aggressive behavior, depression and hopelessness, low stress resistance, higher insight and metacognition ability. Moreover, other factors are also linked to suicide risk in SZ patients: low adherence to non-pharmacological treatment, more typical antipsychotic prescriptions, long-term illness with several exacerbations, loss of faith in treatment, fear of deterioration, higher insight of the illness, increased positive and fewer negative symptoms, higher nicotine consumption and greater duration of untreated psychosis [17, 24, 25].

On the other hand, the study of the clinical characteristics of the DS patients highlights the important role played by cognition. Compared with the single condition, in the DS it has been observed a worse global cognitive state [26] with a marked deterioration with age, especially in executive performance [2, 3, 27], and it is considered to be a powerful predictor of maladaptive functioning [26]. Interestingly, higher premorbid intelligence quotient [17, 28], better executive [29, 30] and social premorbid functioning [18], and dysfunctional impulsivity [9, 19, 28] have been hypothesized to predispose for suicidal behavior and could facilitate the illegal substance acquisition. In this context, the primary drug of dependence seems decisive given that alcohol is the substance that has shown greater neurodegenerative brain effects and cognitive impairment [31].

Taking all the factors stated above into consideration, DS individuals are a vulnerable population to commit suicide, given that they meet many of the preconditions for the emergence of suicidality. However, few studies have evaluated the executive functioning of the DS patients and none has addressed its possible influence on the existence of lifetime suicide attempts and clinical variables that may be modulating results, so new research is needed to clarify these questions.

The aim of this study was to explore, firstly, the executive functioning differences between suicide attempters and non-attempters in DS patients and the possible premorbid and clinical related factors. To do it an exhaustive clinical evaluation and a complete neuropsychological battery was applied, being all patients in remission of SUD and clinically stables.

## Materials and Methods

### Participants

The participants were 50 DS male patients (36.06 ± 7.79 years) from seven medical centers in Barcelona. They were assigned to two groups according to the self-reported previous suicide attempts: DS attempters (DS+; n = 24) and DS non-attempters (DS-; n = 26). Our sample size was aimed to have a balanced design in two groups. Both groups were abstinent for at least three months, controlled by urinalysis.

Patients were derived by medical centers from their diagnoses according to the DSM-IV-TR (2000) [32] and inclusion/exclusion criteria required. However, each diagnosis was confirmed in a first evaluation session by a researcher responsible for clinical assessment (Master in Clinical Psychology) using the SCID-I and a structured interview of sociodemographic and clinical data. Occasionally, some patients were ruled out for not complying with the dual diagnosis required. The inclusion criteria were: (1) current diagnosis of psychotic spectrum disorder (schizophrenia and schizoaffective) and current SUD in remission for at least three months; (2) under treatment; (3) male gender, consistent with the sex prevalence of the disorder; (4) age between 18–55 years; (5) clinically stable psychiatric symptomatology. The exclusion criteria were: (1) DSM-IV-TR criteria for a current substance induced-psychiatric disorder or psychiatric disorder due to a medical condition; (2) other severe medical illness; (3) mental retardation, history of traumatic brain injury or neurological injury; (4) receiving electroconvulsive therapy within 12 months prior to their study participation. From initially derived sample (n = 56) six patients were discarded for failing to meet the diagnostic and clinical criteria. None of the patients included in the study dropped out and they all completed data recording sessions.

All patients were in treatment for their clinical conditions (SUD and psychotic disorder) with an integrated intervention in which addiction and mental health intervention are offered at the same time and by the same team [5]. Integrated intervention includes a combination of motivational interviewing, contingence and case management, cognitive behavioral therapy, social skills training and relapse prevention.

The ethic committee of the University of Barcelona approved this study, which complies with the ethical standards on human experimentation and with the Helsinki declaration of 1975, as revised in 2008. All participants provided written informed consent and were not compensated for their participation. Data collection was carried out between 2013 and 2015.

### Clinical Measures

Information was collected using the Structural Clinical Interview for DSM-IV-TR Axis I Disorders (SCID-I) [33] along with a structured interview of sociodemographic and clinical data. We confirmed the self-reported data with the medical history of the medical centers, with especial emphasis in our classificatory criteria of lifetime suicide attempts. The suicide attempts in DS+ group range from 1 to 5, with the following distribution: 8 patients with one attempt, 8 with two attempts, 5 with three attempts, 1 with four attempts and 2 with five. Chlorpromazine equivalent doses (CPZ) were calculated for antipsychotic medication [34]. We also recorded daily consumption of cigarettes and caffeine (milligrams of coffee, tea or cola), and the Fagerström test [35] was administered to smokers for nicotine dependence.

Additionally, the Clinical Global Impression-Severity scale (CGI-S) [36] was applied as a subjective measure of the clinical severity. The general level of symptoms and functioning was assessed through the Global Assessment of Functioning (GAF) [37]. The Positive and Negative Syndrome Scale (PANSS) [38] was administered as a measure of psychotic symptom severity, and item 12 was used for Insight evaluation. Social adaptation was measured by the Social Adaptation Self-Evaluation Scale (SASS) [39]. Depressive symptoms were assessed with the Beck Depression Inventory (BDI) [40] and the Plutchik Risk of Suicide Scale (RS) [41] was applied to assess the current suicide risk, establishing the cut-off of 6 points [42]. Finally, the severity of the SUD was measured with the Drug Abuse Screening Test (DAST-20) [43].

### Neuropsychological Assessment

We used the Vocabulary and Block Design subtests (WAIS-III) as a reliable estimate of verbal and non-verbal premorbid IQ [44]. To obtain measures of dorsolateral prefrontal function we selected the Backward Digits subtest (WAIS-III), the Trail Making Test -trail B- (TMT-B), the Tower of Hanoi (ToH) and the standard computerized version of the Wisconsin Card Sorting Test (WCST) as measures of working memory, set-shifting, planning abilities and problem solving, respectively. The Iowa Gambling Task (IGT) was administered to measure decision-making capacity, as a measure of dysfunctional impulsivity related to orbitofrontal cortex functioning [45]. Raw scores were transformed to Z scores (mean = 0 ± SD = 1) for all tests (global scores) and its subcomponents, based on the Spanish normative data for the WAIS-III subtests [46], TMT-B [47, 48], WCST [49], on Mexican normative data for ToH [50], and on American normative data for IGT [51]. An average of Z scores (composite summary scores) were conducted to yield a general measure for both premorbid IQ and executive functioning.

### Data Analysis

Group differences in demographic and clinical variables were explored with the Mann-Whitney U test (U) or with the Chi-Square test for categorical variables. If the quantitative data fulfilled the necessary conditions, the Student’s t-test (t) was used; when the conditions were not met, the nonparametric Mann-Whitney U test was used instead. Differences in Z scores of neuropsychological performance between groups were analyzed with one-way analysis of covariance (ANCOVA) for Vocabulary, Block Design, Backward Digits subtests, TMT-B and composite summary scores for both premorbid IQ and executive functioning. Multivariate analysis of covariance (MANCOVA) was conducted in the case of the ToH, WCST and IGT. Repeated measures multivariate analysis of variance (RM MANCOVA) was also used for the five blocks of the IGT direct scores. In all cases, we considered age as covariate to control for possible effects in cognitive performance because, although groups did not show differences, are known the age-related effects both in general population [50] and DS patients [2, 3]. We repeated all significant analyses including alcohol dependence also as covariate. The partial squared Eta (*η*_*p*_^2^) statistic was used to measure the effect size, in conjunction with the observed power through the option available in the analysis of variance, and the post-hoc comparisons were Bonferroni corrected. Finally, we conducted stepwise linear regressions considering only the significant variables (p < 0.05) found in a previous bivariate correlation analysis for executive functioning and current suicide risk. In all cases the conditions of application and non-existence of collinearity and multicollinearity were verified. Data were analyzed using the Statistical Package for the Social Sciences (SPSS; version 21.0).

## Results

### Demographic and Clinical Data

Groups did not differ in sociodemographic data, medical comorbidity, CPZ, CGI, GAF, depressive symptoms, social adaptation, daily caffeine intake and current suicide risk scores (see Table 1). Although no significant difference were found between groups in the suicide risk, considered as present or absent (*χ*^*2*^_(1)_ = 1.82; p = 0.18), we have observed a tendency of higher proportions of patients with suicide risk in the DS+ (84.6%) compared to the DS- group (58.3%). Daily medication was equivalent between groups, except for use of interdictors, with higher consumption in the DS+ group. Moreover, DS+ showed a higher number of first-degree relatives both with SUD and psychiatric disorders, daily cigarette consumption and nicotine dependence compared to DS-. No significant differences were found between groups in psychotic symptoms (PANSS scores).

**Table 1:** Sociodemographic and clinical data of Dual Schizophrenia (DS) patients, and considering the presence (DS+) or absence (DS-) of suicide attempts. Mean, standard deviation, frequency with percentage, and statistical contrast (Student’s t, Mann-Whitney U or Chi-Square test).

	DS (N = 50)	DS- (N = 26)	DS+ (N = 24)	Statistical contrast
**Sociodemographic data**				
Age (yr)	36.06 (7.79)	35.92 (8.63)	36. 21 (6.95)	t_(1,48)_ = 0.12
Marital status				*Χ*^*2*^_(2)_ = 2.48
Single	41 (82.0%)	22 (84.6%)	19 (79.2%)	
Stable partner	3 (6.0%)	2 (7.7%)	1 (4.2%)	
Separated/Divorced	6 (12.0%)	2 (7.7%)	4 (16.6%)	
Years of education	9.84 (2.06)	9.38 (1.94)	10.33 (2.12)	t_(1,48)_ = 1.65
Economic situation				*χ*^*2*^_(4)_ = 2.68
Active	5 (10.0%)	2 (7.7%)	3 (12.5%)	
Disability pension	35 (70.0%)	17 (65.4%)	18 (75.0%)	
Unemployed	5 (10.0%)	3 (11.5%)	2 (8.3%)	
No income	3 (6.0%)	2 (7.7%)	1 (4.2%)	
Off work (on sick leave)	2 (4.0%)	2 (7.7%)	0 (0%)	
**Clinical data**				
Number of medical comorbidities	0.60 (0.81)	0.42 (0.58)	0.79 (0.96)	t_(1,48)_ = 1.64
Respiratory-type ^a^	4 (8.0%)	3 (11.5%)	1 (4.2%)	*χ*^*2*^_(1)_ = 0.92
Metabolic-type ^a^	7 (14.0%)	3 (11.5%)	4 (16.7%)	*χ*^*2*^_(1)_ = 0.27
Infectious-type ^a^	6 (12.0%)	2 (7.7%)	4 (16.7%)	*χ*^*2*^_(1)_ = 0.95
Other ^a^	9 (18.0%)	4 (15.4%)	5 (20.8%)	*χ*^*2*^_(1)_ = 1.43
Daily number of medications	3.20 (1.78)	2.96 (1.93)	3.46 (1.61)	t_(1,48)_ = 0.98
Typical antipsychotics ^a^	13 (26.0%)	7 (26.9%)	6 (25.0%)	*χ*^*2*^_(1)_ = 0.02
Atypical antipsychotics ^a^	45 (90.0%)	22 (84.6%)	23 (95.8%)	*χ*^*2*^_(1)_ = 1.00
Antidepressants ^a^	15 (30.0%)	6 (23.1%)	9 (37.5%)	*χ*^*2*^_(1)_ = 2.33
Mood stabilizers ^a^	12 (24.0%)	6 (23.1%)	6 (25.0%)	*χ*^*2*^_(1)_ = 0.07
Anxiolytics ^a^	21 (42.0%)	9 (34.6%)	12 (50.0%)	*χ*^*2*^_(1)_ = 0.98
Anticholinergics ^a^	13 (26.0%)	8 (30.8%)	5 (20.8%)	*χ*^*2*^_(1)_ = 0.78
Interdictor ^a^	15 (30.0%)	4 (15.4%)	11 (45.8%)	*χ*^*2*^_(1)_ = 5.13*
Other medication ^a^	14 (28.0%)	8 (30.8%)	6 (25.0%)	*χ*^*2*^_(1)_ = 0.64
CPZ equivalent dosage (mg)	41.34 (90.36)	35.32 (97.20)	45.37 (83.53)	t_(1,48)_ = 1.02
Clinical Global Impression	3.84 (1.48)	3.96 (1.40)	3.71 (1.57)	t_(1,48)_ = 0.60
Global Assessment Functioning	65.78 (10.82)	64.46 (10.17)	67.21 (11.52)	t_(1,48)_ = 0.89
Current suicide risk (Plutchik scale)	6.80 (2.41)	5.92 (1.93)	7.62 (2.60)	t_(1,48)_ = 1.18
Depressive symptoms (BDI)	4.53 (4.08)	3.80 (6.34)	4.83 (3.04)	t_(1,48)_ = 0.46
Social adaptation (SASS)	36.55 (9.06)	35.33 (9.92)	38.38 (7.87)	t_(1,48)_ = 0.76
Relatives with psychiatric disorder	0.75 (0.88)	0.48 (0.71)	1.04 (0.98)	t_(1,48)_ = 2.27*
Age of psychotic disorder onset	24.21 (6.70)	23.75 (7.36)	24.67 (6.10)	t_(1,48)_ = 0.47
Duration of illness (yr)	11.88 (7.71)	12.20 (8.95)	11.54 (6.43)	t_(1,48)_ = 0.30
Psychiatric diagnosis				*χ*^*2*^_(1)_ = 0.02
Schizophrenia	42 (84.2%)	22 (85.0%)	20 (83.3%)	
Schizoaffective	8 (15.8%)	4 (15.0%)	4 (16.7%)	
PANSS-Positive symptoms	11.37 (5.79)	10.79 (4.15)	11.83 (6.89)	t_(1,48)_ = 0.53
PANSS-Negative symptoms	14.00 (7.19)	12.57 (6.66)	15.11 (7.57)	t_(1,48)_ = 0.99
PANSS-General psychopathology	30.09 (12.46)	27.43 (11.77)	32.16 (12.91)	t_(1,48)_ = 1.08
Insight	2.10 (1.37)	1.62 (1.04)	2.44 (1.50)	t_(1,48)_ = 1.81
Fisrt degree relatives with SUD				U = 180.00**
None	30	23	7	
One	6	3	3	
Two or more	14	0	14	
Age of intake onset (yr)	17.22 (5.20)	17.64 (5.88)	16.79 (4.47)	t_(1,48)_ = 0.57
Duration of SUD (yr)	18.39 (8.13)	17.97 (9.01)	18.83 (7.28)	t_(1,48)_ = 0.37
Primary drug of dependence				*χ*^*2*^_(3)_ = 1.14
Alcohol	24 (48.0%)	11 (42.3%)	13 (54.2%)	
Cocaine	13 (26.0%)	8 (30.8%)	5 (20.8%)	
Cannabis	10 (20.0%)	5 (19.2%)	5 (20.8%)	
Other	3 (6.0%)	2 (7.7%)	1 (4.2%)	
Number of substances used	3.80 (1.78)	3.31 (1.57)	4.26 (1.85)	t_(1,48)_ = 1.99
Cocaine ^a^	47 (94.0%)	24 (92.3%)	23 (95.8%)	*χ*^*2*^_(1)_ = 0.27
Cannabis ^a^	39 (78.0%)	20 (76.9%)	19 (79.2%)	*χ*^*2*^_(1)_ = 0.37
Alcohol ^a^	41 (82.0%)	17 (65.4%)	24 (100%)	*χ*^*2*^_(1)_ = 10.13**
Psychodysleptics ^a^	22 (44.0%)	10 (38.5%)	12 (50.0%)	*χ*^*2*^_(1)_ = 0.67
Opioids ^a^	12 (24.0%)	4 (15.4%)	8 (33.3%)	*χ*^*2*^_(1)_ = 2.20
Sedatives ^a^	10 (20.0%)	4 (15.4%)	6 (25.0%)	*χ*^*2*^_(1)_ = 0.72
Months of abstinence	6.64 (4.15)	6.50 (4.81)	6.79 (3.40)	t_(1,48)_ = 0.24
Number of relapses				U = 211.50
None	18	11	7	
One	10	7	3	
Two or more	22	8	14	
Severity of SUD (DAST-20)	12.00 (2.62)	12.67 (2.96)	11.43 (2.24)	t_(1,48)_ = 1.21
Intermediate	26.9	25.0	28.6	
Substantial	69.2	66.7	71.4	
Severe	3.8	8.3	0	
Daily number of cigarettes	20.50 (13.49)	16.08 (11.52)	25.29 (14.04)	t_(1,48)_ = 2.54*
Nicotine dependence (Fagerström test)	5.82 (2.77)	5.00 (2.73)	6.71 (2.57)	t_(1,48)_ = 2.28*
Daily caffeine intake (mg)	255.51 (203.67)	217.69 (179.58)	300.00 (219.41)	t_(1,48)_ = 1.45

CPZ = Chlorpromazine; BDI = Beck Depression Inventory; SASS = Social Adaptation Self-Evaluation Scale; PANSS = Positive and Negative Syndrome Scale; SUD = Substance Use Disorder; DAST-20 = Drug Abuse Screening Test.

a Percentages will not equal 100 as each participant may take more than one substance or medication and may have more than one medical comorbidity.

*p < 0.05

**p < 0.01.

Regarding SUD characteristics, polyconsumption was the more frequent pattern in the total sample (72%) and also in both groups (DS+: 83% and DS-: 61%). DS+ patients showed greater alcohol consumption than DS-, although the percentage of patients with the alcohol as primary drug of dependence was not different between groups. Moreover, the two groups did not show differences in length of abstinence, number of relapses, duration of SUD and severity of SUD (DAST-20).

### Executive Functioning

The total sample showed a low performance compared to the normative data in the TMT-B, reaction time in the ToH, and trials to first category in WCST (z scores lower to -1SD). In the other tasks, both in the individual components and global scores, as well as in the composite summary score of executive functioning, performance tended to be worse than average but in no case exceeds -1SD (Table 2).

**Table 2:** Neuropsychological tests data in Z scores of Dual Schizophrenia (DS) patients, and considering the presence (DS+) or absence (DS-) of suicide attempts. Mean, standard error and statistical contrast carried out (ANCOVA or MANCOVA with age as covariate).

Neuropsychological Tests	DS (N = 50)	DS- (N = 26)	DS+ (N = 24)	F_(1,47)_	Effect size	Power
**PREMORBID IQ**						
Vocabulary (WAIS-III)	-0.14 (0.11)	-0.02 (0.15)	-0.27 (0.16)	1.272		
Block Design (WAIS-III)	-0.17 (0.15)	-0.21 (0.20)	-0.13 (0.22)	0.071		
**Composite Summary Score**	-0.16 (0.12)	-0.12 (0.16)	-0.20 (0.17)	0.130		
**EXECUTIVE FUNCTIONING**						
Working Memory (Backward digits, WAIS-III)	0.03 (0.19)	0.10 (0.26)	-0.10 (0.28)	0.634		
Set-shifting / Cognitive Flexibility (TMT- B)	-1.03 (0.12)	-1.00 (0.18)	-1.07 (0.18)	0.060		
Planning abilities (Tower of Hanoi)						
Number of movements	0.32 (0.14)	0.36 (0.19)	0.28 (0.20)	0.092		
Reaction time	-1.24 (0.20)	-1.21 (0.28)	-1.28 (0.30)	0.031		
Global score	-0.46 (0.15)	-0.42 (0.20)	-0.50 (0.21)	0.065		
Abstract reasoning/Problem solving (WCST)						
Trials	-0.69 (0.16)	-0.37 (0.22)	-1.01 (0.24)	3.755		
Total correct	0.59 (0.16)	0.52 (0.22)	0.66 (0.23)	0.184		
Total errors	-0.34 (0.14)	-0.08 (0.19)	-0.61 (0.20)	3.767		
Perseverative errors	0.41 (0.18)	0.66 (0.26)	0.16 (0.27)	1.777		
Non-perseverative errors	-0.76 (0.12)	-0.53 (0.16)	-1.00 (0.17)	3.898		
Conceptual level responses	-0.49 (0.15)	-0.22 (0.21)	-0.77 (0.21)	3.459		
Categories completed	-0.74 (0.24)	-0.05 (0.34)	-1.42 (0.35)	8.013**	0.146	0.792
Trials to first category	-1.12 (0.34)	-0.36 (0.47)	-1.88 (0.49)	4.969*	0.096	0.598
Failure to maintain set	-0.69 (0.20)	-0.43 (0.28)	-0.96 (0.29)	1.678		
Learn to learn	-0.24 (0.21)	0.15 (0.28)	-0.63 (0.30)	3.631		
Global score	-0.43 (0.13)	-0.06 (0.18)	-0.80 (0.19)	7.940**	0.145	0.788
Decision-making (Iowa Gambling Task)						
Block 1 (trials 1–20)	0.40 (0.10)	0.55 (0.13)	0.25 (0.14)	2.744		
Block 2 (21–40)	-0.28 (0.08)	-0.26 (0.11)	-0.31 (0.12)	0.098		
Block 3 (41–60)	-0.59 (0.10)	-0.32 (0.14)	-0.87 (0.15)	7.790**	0.142	0.781
Block 4 (61–80)	-0.66 (0.12)	-0.37 (0.17)	-0.95 (0.17)	5.636*	0.103	0.651
Block 5 (81–100)	-0.57 (0.11)	-0.21 (0.15)	-0.93 (0.16)	10.457**	0.182	0.886
Total score (100 trials)	-0.47 (0.09)	-0.18 (0.12)	-0.76 (0.13)	11.249**	0.193	0.907
**Composite Summary Score**	-0.48 (0.07)	-0.31 (0.10)	-0.65 (0.10)	5.699*	0.105	0.685

IQ = intelligence quotient; WAIS-III = Wechsler Adult Intelligence Scale-Revised Third Edition; TMT-B = Trail Making Test, trail B; WCST = Wisconsin Card Sorting Test

* p < 0.05

**p < 0.01.

The groups showed similar premorbid IQ, considering both the verbal component as the manipulative, and only provided differences in the performance of WCST and IGT. The WCST analyses showed significant worse scores for the DS+ patients in categories completed, trials to first category and in the global measure of WCST performance. However, these differences disappeared when the alcohol dependence was considered as a covariate in the analyses (F _(1,46)_ < 2.229; p > 0.143; *η*_*p*_^2^ < 0.049).

**Fig 1:**
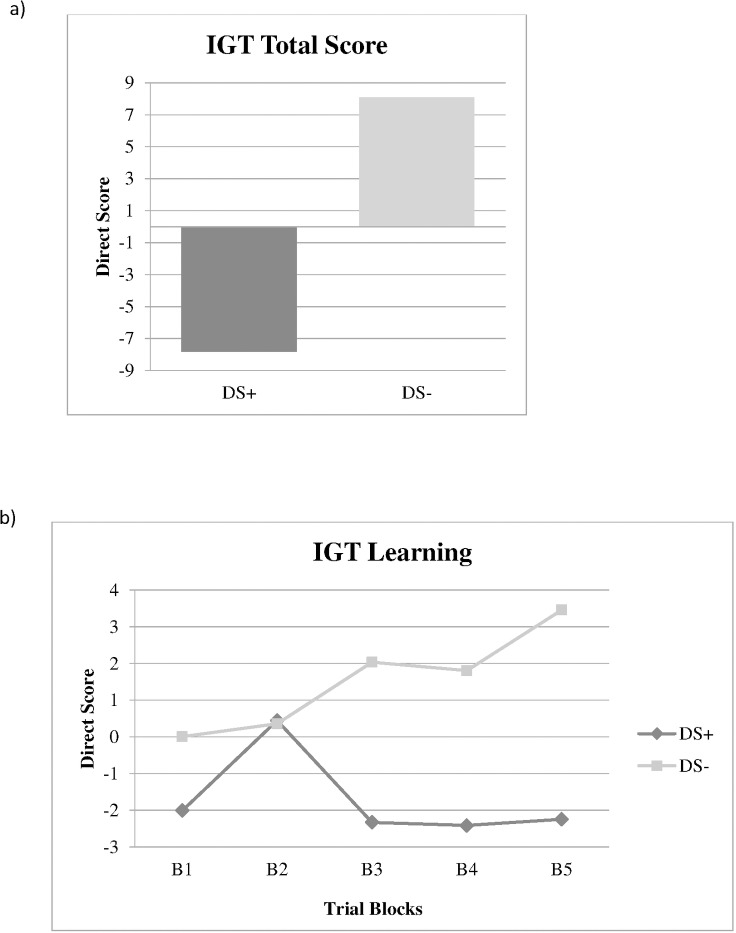
**Total score (100 trials) (a) and learning (b) in Iowa Gambling task (IGT) for Dual Schizophrenia patients with (DS+) and without (DS-) suicide attempts.** B1: trials 1–20; B2: trials 21–40; B3: trials 41–60; B4: trials 61–80; B5: trials 81–100.

Considering the IGT, we obtained significant differences in the third, fourth and last block trials as well as in the total score (100 trials), with the DS+ group showing the worst performance. Although the Z scores for both groups in the IGT were within the mean, when the total direct scores were considered regarding the type of decision-making chosen, the DS+ group showed a disadvantageous pattern in comparison to the DS- (F _(1,47)_ = 11.274; p = 0.002; *η*_*p*_^2^ = 0.193; power = 0.907) (see Fig 1A). Similarly, the RM MANCOVA for the direct scores showed a significantly worse performance of the DS+ group (F _(1,47)_ = 5.203; p = 0.027; *η*_*p*_^2^ = 0.100; power = 0.636), with a negative learning compared to the DS- (see Fig 1B). All significant differences between groups in the IGT remained when alcohol dependence was considered as a covariate in the analyses (F _(1,46)_ > 4.64; p < 0.037; *η*_*p*_^2^ > 0.092; power > 0.562).

Finally, the composite summary score of executive functioning was worse in DS+ group as compared to DS- and it continued providing significant differences when in the analysis the alcohol dependence was included as covariate (F _(1,46)_ = 4.982; p = 0.032; *η*_*p*_^2^ = 0.096; power = 0.603).

### Factors Related to Executive Functioning and Current Risk of Suicide

In the total sample, the regression analysis indicated that the model was significant for all cognitive tasks except the IGT, and also for the composite summary score of executive functioning (see Table 3). The significant independent variables related to the different task were premorbid IQ, GAF, negative symptoms (PANSS), severity (DAST-20) and duration of SUD. Premorbid IQ explained 39% of the variance of working memory task and, together with duration of SUD, 57% of the variance of WCST. Execution is better as higher OQ and shorter duration of SUD. Furthermore, GAF explained 13% of the variance in the Backward Digits subtest, being the execution higher as better global functioning. Negative symptoms with DAST-20 described 24% of the variance of ToH test, patients with less negative symptoms and higher severity of addiction performed better. Considering the executive functioning (composite score), premorbid IQ, DAST-20 and duration of SUD accounted for 52% of their variance. The best composite scores of executive functioning are related to higher IQ and severity of addiction and shorter duration of SUD.

**Table 3:** Stepwise linear regression analysis of each independent variable, explaining executive functioning of Dual Schizophrenia (DS) patients, and considering the presence (DS+) or absence (DS-) of suicide attempts.

Executive functioning	Adjusted R^2^	F	Independent variables ^a^	β Standardized	p values	Condition index^b^
**DS (N = 50)**						
Working Memory (Backward digits, WAIS-III)	0.393	30.464	Premorbid IQ	0.627	0.001	1.205
Set-shifting / Cognitive Flexibility (TMT- B)	0.127	7.952	GAF	0.380	0.007	12.281
Planning Abilities (ToH)	0.241	4.813	Negative symptoms (PANSS)	-0.474	0.015	10.800
			Severity of SUD (DAST-20)	0.397	0.049	
Abstract Reasoning / Problem Solving (WCST)	0.574	9.092	Premorbid IQ	0.499	0.001	4.880
			Duration of SUD (yr)	-0.606	0.002	
Composite summary score	0.522	6.014	Premorbid IQ	0.592	0.006	10.804
			Severity of SUD (DAST-20)	0.581	0.008	
			Duration of SUD (yr)	-0.505	0.028	
**DS- (N = 26)**						
Working Memory (Backward digits, WAIS-III)	0.465	22.758	Premorbid IQ	0.698	0.001	1.550
Set-shifting / Cognitive Flexibility (TMT- B)	0.305	6.274	Months of abstinence	0.515	0.006	4.008
			SUD relapses	0.378	0.041	
Planning Abilities (ToH)	0.483	5.532	Severity of SUD (DAST-20)	0.875	0.008	7.570
Abstract Reasoning / Problem Solving (WCST)	0.522	6.996	Premorbid IQ	0.447	0.014	4.390
			Duration of SUD (yr)	-0.751	0.006	
Composite summary score	0.872	12.311	Premorbid IQ	0.781	0.019	10.168
			Severity of SUD (DAST-20)	0.957	0.005	
			Duration of SUD (yr)	-0.499	0.041	
**DS+ (N = 24)**						
Working Memory (Backward digits, WAIS-III)	0,260	8.713	Premorbid IQ	0,542	0.008	1.267
Set-Shifting / Cognitive Flexibility (TMT- B)	0.466	10.583	Duration of SUD (yr)	-0.741	0.001	6.398
			Premorbid IQ	0.682	0.001	
Planning Abilities (ToH)	0.380	4.676	Negative symptoms (PANSS)	-0.760	0.012	4.245
Abstract Reasoning / Problem Solving (WCST)	0.356	7.968	Premorbid IQ	0.524	0.010	6.305
			Duration of SUD (yr)	-0.436	0.016	
Composite summary score	0.517	4.209	Premorbid IQ	0.726	0.014	6.195
			Duration of SUD (yr)	-0.465	0.029	

WAIS-III = Wechsler Adult Intelligence Scale-Revised Third Edition; IQ = Intelligence Quotient; TMT-B = Trail Making Test, trail B; GAF = Global Assessment of Functioning; ToH = Tower of Hanoi; PANSS = Positive and Negative Syndrome Scale; SUD = Substance Use Disorder; DAST-20 = Drug Abuse Screening Test; WCST = Wisconsin Card Sorting Test; IGT = Iowa Gambling Task.

^a^ Only significant variables are presented that comprise each explicative model.

^b^. In models with more than one variable is included the superior.

In all cases, the Tolerance values were higher than 0.815 and the Variance Inflation Factor values lower than 1.209.

In the DS- group, similarly to the total sample, premorbid IQ was significant for Backward Digits subtest, explaining 47% of the variance and, together with duration of SUD described 52% of the variance of WCST. In both cases higher IQ was related to better performance, and in the second case with the shorter duration of SUD. On the other hand, DAST-20 described 48% of the variance of ToH test and months of abstinence and SUD relapses accounted for 31% of the variance in TMT-B. The planning ability is better as higher severity of addiction and the superior cognitive flexibility as more months of abstinence and lower relapses. Considering the DS+ group, also premorbid IQ explained the variance in Backward Digits subtest, in this case 26%. As higher IQ, the better the performance in working memory. Additionally, IQ together with duration of SUD explained 47% of the variance in TMT-B and 36% of the variance in WCST. In both cases, as higher is the IQ better is the execution and as shorter duration of SUD better cognitive flexibility. Finally, negative symptoms described 38% of the variance in ToH task, as higher presence of symptoms worse the performance. For the executive functioning (composite score), in the DS- group premorbid IQ, DAST-20 and duration of SUD explained the 87% of its variance, while in the DS+ group IQ together with duration of SUD accounted for the 52% (see Table 3). In all cases the sense of the relationship is established as described in the total sample.

We found significant correlations between current suicide risk and number of first-degree relatives with SUD and insight, which accounted for 64% of the variance for the total sample. When considering the groups this result correspond to the DS+ patients, with first-degree relatives with SUD and insight together explaining the 87% of their variance for current suicide risk. In contrast, in DS- group the 47% of the variance of current suicide risk was explained for the positive symptoms (PANSS) (Table 4).

**Table 4:** Stepwise linear regression analysis of each independent variable explaining current suicide risk of Dual Schizophrenia (DS) patients, and considering the presence (DS+) or absence (DS-) of suicide attempts.

Suicide risk *(Plutchik scale)*	Adjusted R^2^	F	Independent variables ^a^	β Standardized	p values	Condition index^b^
**DS** (N = 50)	0.644	18.090	First-degree relatives with SUD	0.601	0.001	4.199
			Insight (PANSS)	0.413	0.007	
**DS-** (N = 26)	0.467	10.650	Positive symptoms (PANSS)	0.718	0.009	5.916
**DS+** (N = 24)	0.870	37.747	First-degree relatives with SUD	0.718	0.001	5.019
			Insight (PANSS)	0.442	0.007	

SUD = Substance Use Disorder; PANSS = Positive and Negative Syndrome Scale.

^a^ Only significant variables are presented that comprise each explicative model.

^b^ In models with more than one variable is included the superior.

In all cases, the Tolerance values were higher than 0.890 and the Variance Inflation Factor values lower than 1.124.

## Discussion

To our knowledge, this is the first study aiming to explore the executive functioning, current suicide risk and clinical state in DS patients, considering the presence or absence of lifetime suicide attempts and related factors.

We did not find differences in any sociodemographic variables or in most of the clinical ones between DS+ and DS- patients. The only differences observed were that the DS+ group showed a higher number of daily cigarette intake and more nicotine dependence, greater rates of alcohol consumption and use of interdictors, which may be due to the need to relieve the dysphoric mood in these patients [24, 52, 53].

The groups neither showed significant differences in current suicide risk scores, although we observed more patients in DS+ than in DS- group fulfilling the risk criteria. Regarding pharmacological treatment, the literature suggests that typical antipsychotics are more related to suicidality due to their side effects, while atypical antipsychotics reduce the rates of attempts [54]. However, and in line with other authors [9], we did not find differences in typical or atypical antipsychotic prescriptions between groups, or in CPZ. Furthermore, we did not find differences in GAF or CGI-S scores, depressive or psychotic symptoms or in social adaptation, suggesting similar psychosocial functioning in both groups at the moment of assessment. The good clinical condition of patients and the similarity between groups is due to the schizophrenia stabilization as well as to SUD treatment in which is worked therapeutically the presence of psychiatric symptomatology by means of an integrated treatment which seems advantageous compared to specific interventions in DS [5]. The absence of differences between groups in clinical variables, which is known may influence the cognitive performance, supports the validity of our results in relation to lifetime suicide attempts and executive functions. It is possible that the consideration of patients with less demanding treatments and less abstinence time contributes to greater differences between groups, which should be explored in future studies.

Although precedent studies about neurocognition in DS are heterogeneous, it has been observed that DS patients are characterized by a worse global cognitive state [26], which is age-related, especially in executive performance [2, 3, 27] compared to single SZ. Regarding lifetime suicide attempts, in some studies that pointed out a better functioning in SZ patients with this condition related to the ability to initiate and plan suicidal behavior [29, 30], little information about the comorbidity with SUD was considered. In our study, while DS patients showed relative good global executive performance compared to normative data, they presented a specifically impaired functioning in set-shifting, planning abilities and problem solving skills. On the other hand, when groups are considered, DS+ patients showed worse general executive functioning and low performance for both problem solving skills and disadvantageous decision-making, without differences in premorbid IQ. Our results suggest that previous life time suicide attempts are related to executive deficits, although we cannot discard that other general cognitive deficits (i.e. attention) are also involved. In line with prior studies [9, 19, 28], these results suggest that the previous lifetime suicide attempts could increase the dysfunctional impulsivity. Nevertheless, as it has been previously described [20, 31], we found that alcohol consumption accounts for the problem solving skills impairment. Thus, it seems essential to consider the main substance of dependence for a better limitation of the executive functioning in DS and the impact of lifetime suicide attempts on it.

We want to note that the lack of differences in executive functioning in a SZ sample with or without lifetime suicide attempts found in some studies [9, 55] may be due to the low sensitivity of the neuropsychological tasks used. In contrast, the IGT and WCST used in our study could be good measures to elucidate impairments in both orbitofrontal and dorsolateral functioning in patients with lifetime suicide attempts, although the influence of alcohol dependence in problem solving needs to be further explored. Furthermore, in our sample, the DS+ group had more first-degree relatives with SUD or a psychiatric illness, suggesting there may be an influence of genetic liability or social environmental factors in suicide attempts [17].

Moreover, several factors modulate executive performance of DS patients. Thus, the severity of SUD seems to play a positive influence on executive functioning and specifically in planning abilities, in contrast to negative symptoms. The result of the DS- group indicates that this is probably due to the necessity to obtain substances to maintain drug use in subjects with a stronger addiction [9, 28, 31], which could somehow mitigate their negative affectivity. Moreover, patients with greater severity of SUD require often treatment younger than those with less severity and, therefore, their cognitive functioning could be more preserved of the neurotoxic damage associated with the consumption [3, 31]. However, the results in the DS+ group suggest that negative symptoms may hinder planning for achieving life goals and quality of life [56], which in previous studies has been associated with suicide attempts [18]. However, in the DS- group we found the positive symptoms associated with the current risk suicide, possibly due to an increased impulsivity [9, 17, 27]. Furthermore, in DS patients the duration of SUD was related to cognitive impairment on general executive performance and with problem solving and set-shifting abilities [6] and the poor psychosocial functioning was related to set-shifting difficulties, which could increase the difficulty of rehabilitation [26]. It is noteworthy that the premorbid IQ could be a crucial neuroprotector factor [50] for working memory, problem solving and global executive functioning of DS patients, independently of lifetime suicide attempts, as well as for cognitive flexibility in DS+. On the other hand, in the DS- group better set-shifting performance appears related to both longer abstinence period and higher SUD relapses. In this way, the assessment of executive functions and the consideration of clinical risk factors for integrating cognitive remediation therapy could be beneficial for the effectiveness of treatment and clinical course of DS patients [26], especially for those DS+ or with suicide risk, similarly to that hypothesized in SZ [57], and could be determinant for a better prognosis in social and occupational function.

Our results highlight the importance for the greater suicide risk score of having more insight and first-degree relatives with SUD in DS+, suggesting the hypothesis that these patients are more vulnerable to the perception of stigma, which increases the future probability of suicide attempts [14, 29]. Even though we have addressed this current knowledge gap in DS individuals, some limitations should be taken into account when interpreting our results: (i) The suicide attempts were collected retrospectively and self-reported by patients, without recorded the method of attempted and its seriousness. Although we contrasted this information with medical records and their treating psychiatrist, we do not exclude that such information might be biased; (ii) The clinical and neuropsychological assessment took place after the individuals had intended suicide; and the results must be understood as associated factors with suicidality; (iii) Our sample is comprised by a relatively small number of patients although higher than most previous studies with DS patients, who often willing to drop out from studies or declining to participate in them. Moreover, the analysis with multiple comparisons without corrections also could increase the probability of false positive results and the low power in some analyses, especially when considering the alcohol dependence, could increase the probability of false negative results. Controlling for diverse confounding variables with our inclusion/exclusion criteria as well as the exhaustive clinical and cognitive evaluation reinforce the obtained results, although future research is needed to overcome the limitations of this study.

## Conclusions

Our results emphasize the importance of taking into consideration the clinical and cognitive factors that modulate the suicidality in DS, in order to prevent future suicide attempts and to develop targeted interventions to reduce such risk, although further studies are needed to provide more consistent conclusions. The DS+ group presented worse general executive functioning, decision-making abilities and problem solving skills, although the last one was influenced by alcohol consumption. Several clinical factors, such as global psychosocial functioning, negative symptoms, SUD characteristics (severity, duration, abstinence period and relapses), and premorbid IQ modulated executive functioning in DS patients. Furthermore, first-degree relatives with SUD, insight scores and positive symptoms were positively associated with suicide risk scores. Our results can be not generalized, having to be taken with caution since it is the first study made in this line and it is not exempt of some methodological limitations. Future works should examine the therapeutic benefit of including neurocognitive remediation therapy when necessary in the integrated treatment of DS patients.
